# How contaminated with ammunition-derived lead is meat from European small game animals? Assessing and reducing risks to human health

**DOI:** 10.1007/s13280-022-01737-9

**Published:** 2022-05-10

**Authors:** Deborah J. Pain, Rhys E. Green, Mark A. Taggart, Niels Kanstrup

**Affiliations:** 1 Department of Zoology, University of Cambridge, Downing Street, Cambridge, CB2 3EJ UK; 2 School of Biological Sciences, University of East Anglia, Norwich Research Park, NR4 7TJ UK; 3 Centre for Conservation Science, RSPB, The Lodge, Sandy, SG19 2DL Bedfordshire UK; 4 Environmental Research Institute, University of the Highlands and Islands, Castle Street, Thurso, KW14 7AP UK; 5 Department of Ecoscience, Aarhus University, C.F. Møllers Allé 8, 8000 Aarhus C, Denmark

**Keywords:** Europe, Game, Human health risk assessment, Lead gunshot, Meat, Regulation

## Abstract

Small game animals are generally hunted with lead gunshot which often fragments causing elevated lead concentrations in meat and presenting health risks to frequent consumers and vulnerable groups. We reviewed three decades of European data on lead concentrations in the meat of gamebirds, rabbits and hares across countries with various restrictions on lead gunshot use. Mean meat lead concentrations decreased to a low level in Denmark, the only country in the study with a total ban on lead gunshot use. In contrast, elsewhere in Europe meat lead concentrations increased over time, an unexplained and previously undescribed finding. The only regulatory restriction associated with a decrease in meat lead concentrations was the Danish total ban on lead gunshot use. We calculated an arithmetic mean lead concentration in small game meat of 5.205 ppm w.w. (2011–2021) from across Europe. EU and UK regulators are considering banning lead ammunition for all hunting to protect human and wildlife health. The mean value we found for small game meat (2011–2021) was fourteen times higher than that used in a recent EU-wide risk assessment.

**Supplementary Information:**

The online version contains supplementary material available at 10.1007/s13280-022-01737-9.

## Introduction

Lead (Pb) is a highly toxic metal with no known biological function which impairs nervous, cardiac, renal, immune, endocrine, and other functions in humans (EFSA 2010; ACCLPP 2012). Brain development in children, as measured by Intelligence Quotient (IQ), is of particular concern, with apparently irreversible effects associated with even low blood lead concentrations. There may be no minimum blood lead threshold below which there is no effect of lead on intellectual function in children (ACCLPP 2012). Consequently, it is important to reduce or eliminate exposures to lead in childhood.

As knowledge of the effects of chronic low-level exposure to lead has increased, governments have progressively regulated to reduce exposure to lead from many anthropogenic sources including paint, petrol, drinking water, and some foods (Stroud 2015; Directive (EU) 2020/2184 (EU 2021)). Today, the primary route of exposure to lead in the European Union (EU) is through the diet. With a view to protecting public health, the European Commission set 0.100 ppm w.w. (wet weight) as the maximum level (EUML) for lead in marketed meat (muscle tissue) from domestic animals, i.e. cattle, sheep, pigs and poultry (Regulation EC1881/2006 (EC 2006)). No EUML has been set for wild game meat, but EU countries report on lead levels in wild game as part of surveillance under Council Directive 96/23/EC (EC 1996) on residues in animal products, using a similar reporting threshold.

European hunters primarily shoot small game animals, such as gamebirds and lagomorphs (rabbits and hares), using lead shotgun ammunition (gunshot) and large game animals, such as deer, with lead bullets. In many countries, wild-shot game meat is supplied to domestic or international markets (Thomas et al. 2020). Lead bullets and gunshot frequently fragment on impact with quarry leaving lead particles widely dispersed across the edible tissue (Hunt et al. 2009; Pain et al. 2010). Many lead particles are too small to be seen or detected in the mouth, so it is not practical to remove them, even with careful butchery, and their ingestion is unavoidable (Pain et al. 2010).

Lead concentrations in muscle tissue from game animals can comprise both biologically incorporated lead and shot-in (embedded) lead. Muscle lead concentrations in wild birds and small mammals that are not lead poisoned and have not been killed using lead gunshot are low, usually below 0.100 ppm w.w. (e.g. Koréneková et al. 2008; Damerau et al. 2013; Hutařová et al. 2013). Biologically incorporated lead consists of lead which has been absorbed into the bloodstream, e.g. via the diet, and then deposited in tissues. Concentrations of biologically incorporated lead tend to be homogenous across a tissue type, with the highest soft tissue lead concentrations found in the liver and kidney and far lower levels in muscle tissue. Nonetheless, when birds are experimentally dosed with lead shot or die from lead poisoning, muscle lead concentrations can reach several ppm w.w. (e.g. Longcore et al.^1974^; Fimreite 1984; Gasparik et al. 2012). In contrast, shot-in lead results in highly heterogenous and often extremely elevated muscle lead concentrations (Scheuhammer et al. 1998), sometimes reaching tens or hundreds of ppm w.w. (Pain et al. 2010; this paper).

Lead concentrations in game animals shot with lead ammunition frequently exceed the EUML of 0.100 ppm w.w. set for meat from domesticated animals (Pain et al. 2010; Gerofke et al. 2018). At least some of this lead is bioaccessible and bioavailable (Hunt et al. 2009; Mateo et al. 2011; Green and Pain 2012) and, therefore, of potential concern for human consumers. A number of national agencies have assessed potential risks to human health from eating meat from wild game shot with lead ammunition (e.g. Spain, AESAN. 2012; UK, FSA 2012; Norway, Knutsen et al. 2015; France, ANSES 2018; Germany, Gerofke et al. 2018; Sweden, Livsmedelsverket 2021). Associated consumer advice generally recommends that frequent consumers reduce the amount of wild game eaten, and that pregnant women and children minimise or avoid consumption of such meat.

The European Chemicals Agency (ECHA) implements the EU’s chemicals legislation to protect human health and the environment. Recognising the risks from lead in ammunition to wildlife, the environment and human health, the European Commission requested that ECHA prepare a proposal to restrict the placing on the market and use of lead in ammunition under the EU Regulation on the Registration, Evaluation, Authorisation and Restriction of Chemicals (EU REACH) (EC 2019). The proposal was published in March 2021 (ECHA 2021) and is now the subject of consultation and review. If accepted, it could be adopted as soon as 2023. The UK Government is considering similar legislation under the UK REACH regulation with a comparable timeline (Defra 2021). These regulatory processes will involve detailed assessments of the risks to human health, the environment and wildlife of lead gunshot use, and the efficacy of risk-reduction measures.

Some EU countries already have measures in place to restrict the use of lead gunshot. These vary considerably, from no restrictions, to voluntary restrictions, partial statutory restrictions, and, in a few cases, total bans on lead gunshot use. An example of a partial ban is that in England, where the use of lead gunshot is prohibited for hunting waterfowl, or over certain wetlands (HMSO 1999), but lead is allowed for hunting terrestrial gamebirds. An example of a total ban is that in Denmark, where the sale, use, and possession of lead gunshot have been prohibited for all purposes since 1996 (Mateo and Kanstrup 2019).

We reviewed publications from the last approximately 30 years that examined muscle lead concentrations in small game animals harvested by European hunters to (1) provide supporting information for an accurate evaluation of the health risks to consumers and (2) determine the effectiveness of existing voluntary and regulatory measures at reducing the amount of ammunition-derived lead in this meat. This review is of particular relevance to the ongoing EU and UK REACH restriction processes for lead in ammunition.

## Methods

### Lead concentrations in small game animals in Europe

*Literature review:* We reviewed the literature, predominantly peer reviewed, between 1990 and 2021 to identify publications that reported arithmetic mean lead concentrations in muscle samples from wild-shot gamebirds and small mammals in Europe. We define small game animals as species with mean body weights < 10 kg. In Europe, the largest frequently shot small game animal is the European hare (*Lepus europaeus*: mean body weight 4.3 kg (Wilson et al. 2016)). Data were compiled and used to compare mean lead concentrations across species of game animal, countries and time periods, and in countries with and without legal restrictions on the use of lead gunshot. Data were used to calculate an overall mean lead concentration for small game for human health risk assessment purposes and to identify the effect of risk-reduction measures on lead concentrations in game meat.

The review was conducted following guidelines set out by the RepOrting standards for Systematic Evidence Syntheses in environmental research (ROSES). Full details of our search strategy, papers identified, inclusion/exclusion criteria and data treatment are given in SI Appendix S1.

*Grey literature:* Lead concentrations in the meat of red grouse (*Lagopus lagopus*; *n* = 40) and pheasant (*Phasianus colchicus*; *n* = 75) from the UK have been published previously, but not peer reviewed (Avery 2016; Wild Justice 2021). All chemical analysis of these samples was undertaken at the Environmental Research Institute (ERI) under the supervision of one of us (MAT). We provide full details of sample collection, preparation and analysis of these samples in SI Appendix S2.

### Risk reduction measures

Various national restrictions on the use of lead gunshot in European countries were considered and include:

1. Statutory measures restricting the use of lead gunshot for shooting waterfowl and/or shooting over wetlands, implemented in different countries at different times.
2. A total ban on the use of lead gunshot for shooting in Denmark in 1996, with an additional information campaign to encourage compliance initiated in 2008.
3. A five-year voluntary transition to the use of non-lead gunshot for the taking of live quarry announced by major shooting organisations in February 2020 in the UK.

We compare mean lead concentrations in muscle tissue from small game between periods before and after such restrictions and between countries with and without restrictions. Additional details are given in SI Appendix S3.

### European food safety authority (EFSA) data

EU-wide data on tissue lead concentrations in small game were provided by EFSA (2020) to ECHA (2021) and used for human health risk assessment (Table 1–43 in ECHA 2021). The EFSA data presumably resulted from EU-wide surveillance, although data collection methods were not stated in ECHA (2021). One of us (DJP) requested details from EFSA in November 2021, but these had not been received at the time of writing (March 2022). Hence, we have no specific information on the countries covered by the EFSA study or the sources of the animals sampled. Consequently, these data have only been used for comparative purposes and not included in our modelling or calculations of means.

### Statistical analyses

We used weighted ordinary least-squares regression models to examine the relationships between log-transformed mean tissue lead concentration and the following explanatory variables: country (16 levels), species/species group (13 levels) and decade (3 levels). We first excluded Denmark as it was the only country for which we had data that had a complete ban on lead ammunition use. Values of ΔAIC_c_ and AIC_c_ weights (Burnham and Anderson 2002) were compared across the eight models fitted. AIC_c_ (small-sample Akaike Information Criterion) is a single number score that helps determine which of a set of models of the same data gives the most parsimonious description of a given dataset. For a given set of models, the one with the lowest AIC_c_ score is usually regarded as the most parsimonious and is often the preferred model (Burnham and Anderson 2002). The AIC_c_ score is lower for models which fit the data values closely, but AIC_c_ has a component which increases in direct proportion to the number of model parameters that are fitted. Hence, the use of AIC_c_ penalises models with many fitted parameters and only selects them if this is justified by a good fit to the data.

Based upon the results of this analysis, we next fitted models using the following variables: species/species group (13 levels), central calendar year of sampling (continuous variable), Denmark/not Denmark (binary), and a two-way interaction term between Denmark or not, and central sampling year of study. We defined the central sampling year of a sampling period as the mean of the first and last calendar years of the period, rounded to the nearest whole number. In addition, we fitted models with Denmark or not, central sampling year, and the interaction term of these two variables, with and without the effect of species.

We calculated weighted arithmetic means and bootstrap confidence intervals with and without Denmark for the whole dataset and different decades. As Denmark and the Netherlands have total bans on lead gunshot use for hunting, we adjusted the mean European lead concentration by using the proportion of game likely to be shot in these countries, by taking the numbers of hunters in these and other European countries as a proxy.

Additional details are given in SI Appendix S4.

## Results

### Lead concentrations in meat from small game animals

Data meeting our selection criteria were available for 13 species/species groups from 16 countries plus one additional study in which various EU countries were combined. One study from Turkey was included in the dataset as part of Turkey falls geographically within Europe. Data were taken from 25 sources. These included 21 peer-reviewed studies; one statutory surveillance report written in the form of a paper with details of sampling and analytical methods (Drápal et al. undated), and the Danish statutory surveillance scheme (DVFA various years). Data are also presented from two UK studies not previously peer reviewed (Avery 2016; Wild Justice 2021; SI Appendix S2). We analysed a total of 76 species-country means. The dataset is provided in SI Appendix S5.

UK studies not previously peer reviewed (Avery 2016; Wild Justice 2021) included data from red grouse (2015) and pheasants (2021) purchased from four retail outlets in England. Results are given in Table 1. Seventy two per cent of samples from these studies exceeded the EUML; 38% by more than an order of magnitude and 6% by more than 2 orders of magnitude. In red grouse from the UK (Table 1), 18 whole shot were found and removed from 14 of 40 carcasses during initial sample preparation. All muscle samples were then subject to X-radiography before lead analysis, and an additional 10 shot were found and removed from 9 birds (7 individuals being different to those in which shot was originally found). Shot was removed prior to analysis as they would normally be removed by consumers at the table.

**Table 1:** Distribution of lead concentrations in muscle tissue of pheasants (2020 and 2021) and red grouse (2015) purchased from 4 retail outlets in the UK

Lead concentrations ppm w.w	Pheasants (*n*)	Red grouse (*n*)	Percentage of samples (combined)
< 0.1	23	9	28
0.1 to < 1.0	23	16	34
1.0 to < 10	24	13	32
10 to < 100	4	1	4
100 to < 1000	0	1	1
≥ 1000	1^a^	0	1
Total	75	40	100

Data from Avery (2016), Wild Justice (2021)

^a^A lead shot was found in this pheasant carcass after it had been prepared for analysis. Because whole shot in game meals are normally discovered and removed at the dinner table, this particular sample was excluded from analysis elsewhere in this paper. Nevertheless, the information has been included as an example of how lead pellets, which are small (a number 6 leadshot is 2.6 mm in diameter), are sometimes missed during visual examination of pheasant carcasses

Eleven additional studies (excluding Denmark) reported the proportion of samples exceeding the EUML, together accounting for 58.1% of samples (292 of 503; and 60.6% when including UK samples, i.e. 374 of 617 samples). Thus, elevated arithmetic mean concentrations do not only result from the occasional very high lead level but also from a high proportion of samples with lead concentrations considerably greater than the EUML.

**Fig. 1:**
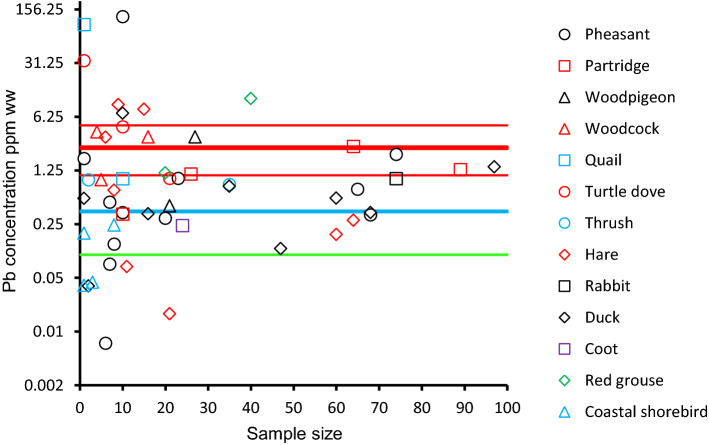
Arithmetic mean lead concentrations (ppm w.w.) in muscle from small game animals in Europe (excluding Denmark) in relation to sample size. The thick red horizontal line shows the grand weighted arithmetic mean concentration based upon all samples (2.474 ppm), with study sample sizes as weights. Thin red lines show the 95% bootstrap confidence interval of this grand mean (1.079 to 4.826 ppm). The blue horizontal line shows the upper bound of the mean for all small game species combined reported by EFSA (2020) (0.366 ppm). The vertical axis is logarithmic, but axis labels show untransformed concentrations. The green horizontal line shows the EUML for lead in meat from domesticated species (0.100 ppm w.w.)

Arithmetic mean muscle lead concentrations from all species/species groups and countries/country groups, excluding Denmark, in relation to the number of samples upon which each mean was based, are presented in Fig. 1. There were no obvious differences in mean concentration among species. There was substantial variation among species-country means when sample sizes were small, but this variation declined markedly with increasing sample size and variation was minor for sample sizes greater than about 70 individuals. The variation at small sample sizes indicated that there were approximately equal proportions of unusually large and unusually small values. The grand weighted arithmetic mean concentration based upon these data (excluding Denmark), with study sample sizes as weights, was 2.474 ppm w.w., with a 95% bootstrap confidence interval of 1.079 to 4.826 ppm w.w. This grand arithmetic mean is somewhat higher than the apparent mean of the plotted symbols in Fig. 1 because the vertical scale of the graph is logarithmic, so the apparent mean of the plotted values is the geometric mean. The arithmetic mean is always larger than a geometric mean based upon the same data by a factor of exp(0.5 V (*n* − 1)/*n*) where *V* is the variance of log_e_-transformed values and *n* is the sample size (Rothery 1988). The upper bound of the arithmetic mean concentration of lead in meat from small game used by ECHA (2021) in their human health risk assessment (upper-bound mean of 0.366 ppm w.w.) is also included in Fig. 1. Our weighted arithmetic mean concentration for data, excluding those from Denmark, was 6.8 times greater than that used by ECHA (2021), and the lower 95% confidence limit of our estimate was 2.9 times larger than the upper-bound mean used by ECHA (2021).

The results from the first phase of weighted ordinary least-squares regression modelling of log-transformed mean concentrations, excluding results from Denmark, indicated that only variation among the three time periods (decades) had an effect on mean lead concentration. There was no indication of substantial effects of species or country. The model including only the effect of time period had an AIC_c_ weight of 1.000, and the model with the next lowest AIC_c_ was the null model and it had a much larger AIC_c_ value (ΔAIC_c_ = 18.69). We, therefore, concluded that effects of species and country on mean lead concentration were minor in this dataset. The fitted model indicated that the geometric mean lead concentration in the third time period (2011–2021) was substantially larger than the geometric means for both 1991–2000 and 2001–2010, which were similar to each other. This conclusion was supported by the weighted arithmetic means for these three periods for countries other than Denmark and their bootstrap 95% confidence intervals (Table 2). We note that the 95% confidence interval for 2011–2021 does not overlap with the confidence intervals for 1991–2000 or 2001–2010. Hence, the arithmetic mean concentration of lead was significantly higher in 2011–2021 than in the earlier periods.

**Table 2:** Weighted arithmetic mean lead concentrations and bootstrap lower (LCL) and upper (UCL) 95% confidence limits in game meat from Europe, excluding and including data from Denmark and during different time periods

Region	Period	Weighted arithmetic mean ppm w.w	95% LCL	95% UCL	*N* studies	*N* samples
Not Denmark	1991–2000	0.747	0.227	1.303	5	250
Not Denmark	2001–2010	0.772	0.411	1.270	22	591
Not Denmark	2011–2021	5.345	1.736	11.482	26	501
Denmark	2001–2010	0.577	0.106	0.804	7	396
Denmark	2011–2021	0.040	0.018	0.065	16	89
All Europe	2011–2021	5.205	1.692	11.180	42	590
Not Denmark	1991–2021	2.474	1.079	4.826	53	1342
Denmark	2001–2021	0.479	0.062	0.761	23	485
All Europe	1991–2021	2.422	1.064	4.708	76	1827

A study is a species/period combination from an individual study; thus, if a published study contains data for two species over the same time period, this counts as two studies

**Fig. 2:**
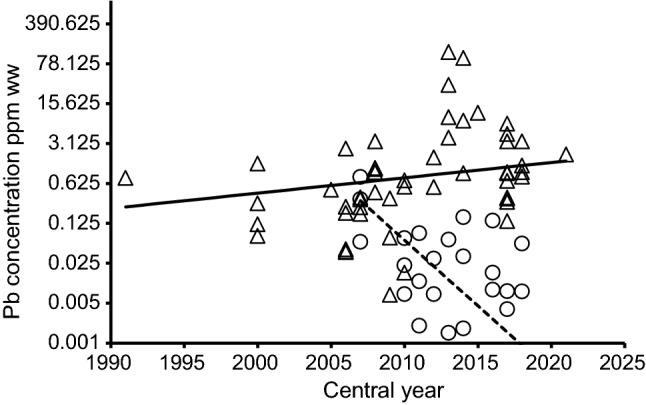
Arithmetic mean lead concentrations (ppm w.w.) in tissue samples from gamebirds from Denmark and small game animals from other European countries in relation to the central year of the sampling period. Results from studies of pheasants, woodpigeons and ducks in Denmark are represented by circles and results from 13 species/species groups of small game from other European countries (including these three) are shown by triangles. Values are plotted in relation to the central year of the sampling period. The lines show expected geometric mean values for the fitted model with the lowest AIC_c_ value. The solid line shows the regression model for countries other than Denmark (log_e_(concentration) = -124.5 + 0.06182*calendar year), and the dashed line shows results for Denmark (log_e_(concentration) = 1062.5 − 0.52998*calendar year). The values on the logarithmic vertical axis are untransformed concentrations

Based upon the results from the first phase of regression modelling, we fitted another set of models to the entire dataset, including results from Denmark, all of which were from sampling periods after the complete ban on the use of lead gunshot was introduced there in 1996. These models examined effects of species, whether or not the study was conducted in Denmark, the central calendar year of the sampling period and the two-way interaction between the last two variables. In the set of ten models compared, the model including the effect of whether the country was Denmark or not, the central year of the sampling period, and the interaction term between these two variables had an AIC_c_ weight of 1.000 and was, therefore, selected as the most appropriate model. The model with the next lowest AIC_c_ included these same variables and the interaction and also the effect of species. However, this model had a much larger AIC_c_ value (ΔAIC_c_ = 17.49). We, therefore, concluded that there were strong effects of central sampling year on geometric mean lead concentration, but the effect of central sampling year differed between Denmark and countries other than Denmark. This difference is illustrated in Fig. 2, which shows the fitted model with the lowest AIC_c_ value. This model indicated that the geometric mean lead concentration increased substantially and significantly with increasing central sampling year for countries other than Denmark, as expected from the results for the first phase of regression modelling, described above (t test on the weighted regression coefficient for countries other than Denmark, *t*_*51*_ = 2.61, *P* = 0.011). By contrast, lead concentration decreased significantly over time in Denmark (*t*_*21*_ = 5.05, *P* < 0.001). The difference in regression slope between Denmark and other countries was statistically significant (*t*_*72*_ = 5.00, *P* < 0.001). This difference in trend between Denmark and the other countries is supported by a comparison among the arithmetic mean lead concentrations for different time periods (Table 2). As described above, period means increased substantially between 2001 and 2010 and 2011–2021 for countries other than Denmark, but the period mean for Denmark in 2011–2021 was much lower than that for 2001–2010 and the 95% confidence intervals for the means for these two periods did not overlap. In the period 2011–2021, the 95% confidence intervals for the arithmetic mean concentrations for Denmark and countries other than Denmark did not overlap (Table 2).

The mean value for small game used by ECHA (2021) in their risk assessment (upper-bound mean of 0.366 ppm w.w.), which presumably comes from recent data, is 14 times lower than the grand weighted arithmetic mean calculated from all published European data (including Denmark) from 2011–2021 (Table 2) and is 4.6 times lower than the lower 95% confidence limit of our arithmetic mean for the whole of Europe.

### The impact of risk-reduction measures on lead concentrations in game meat

**Fig. 3:**
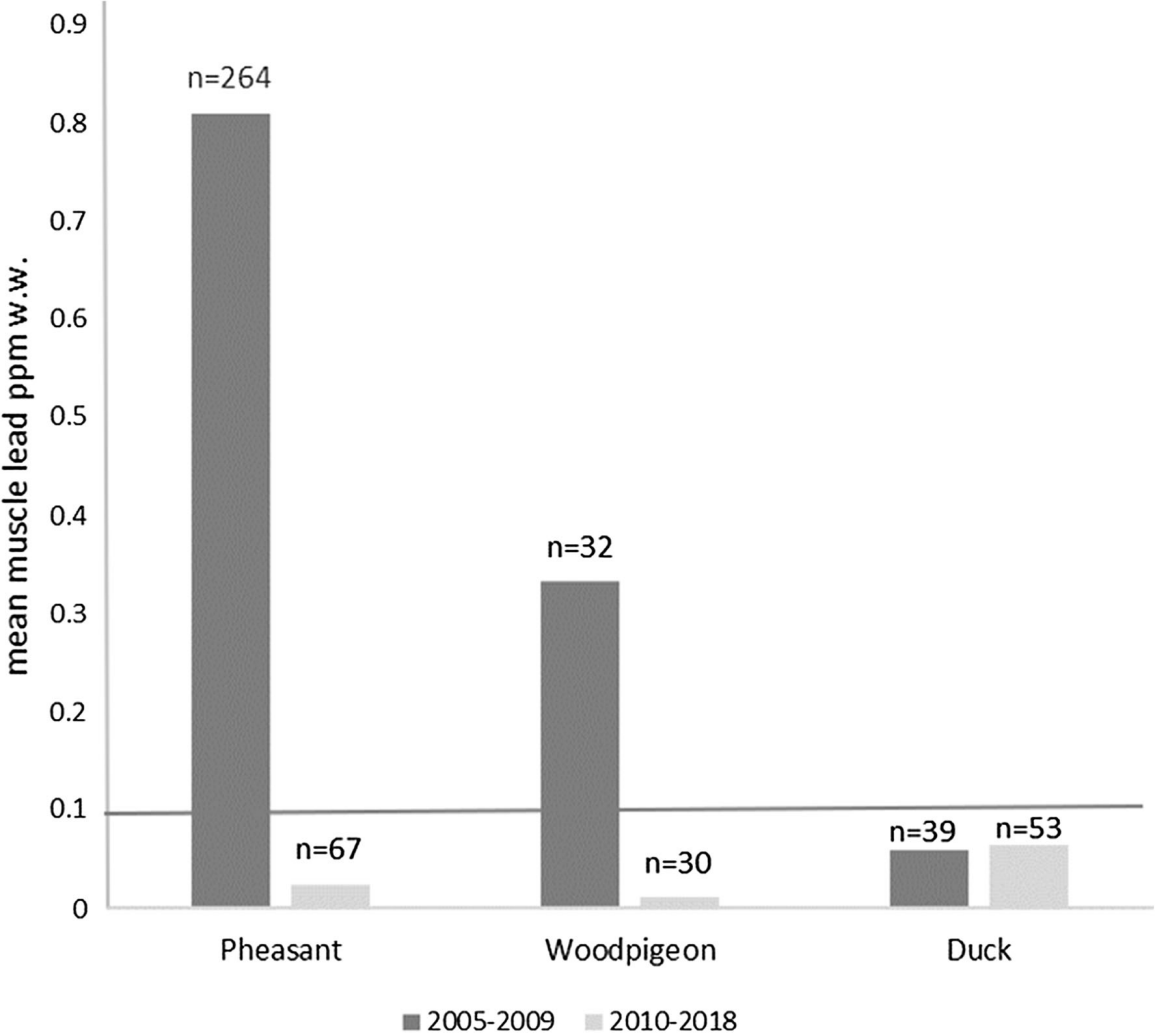
Arithmetic mean lead concentrations in pheasants, woodpigeons and ducks in Denmark measured as part of national surveillance from (1) 2005–2009 and (2) 2010–2018 following an information campaign initiated in 2008 to raise the awareness of hunters about the legislation. Data from DVFA (various). Horizontal line represents the EUML of 0.100 ppm w.w. for meat from domesticated animals

The analysis described above indicates that the mean concentration of lead in meat from small game animals was substantially lower in Denmark than in other countries during the time since a complete ban on the use of lead gunshot was in place in Denmark. None of the other countries for which we had data had a complete ban. Results from national surveillance in Denmark during the period covered by the complete ban are shown separately for three species or species groups in Fig. 3. Although the complete ban was introduced in 1996, the arithmetic mean concentration of lead was substantially larger than the EUML for pheasants and woodpigeons (*Columba palumbus*) during 2005–2009. Only for ducks was the mean concentration lower than the EUML during this period. An awareness campaign was initiated in 2008 to highlight the regulations. In the period 2010–2018, the arithmetic mean concentration of lead was lower than the EUML for all three species.

**Fig. 4:**
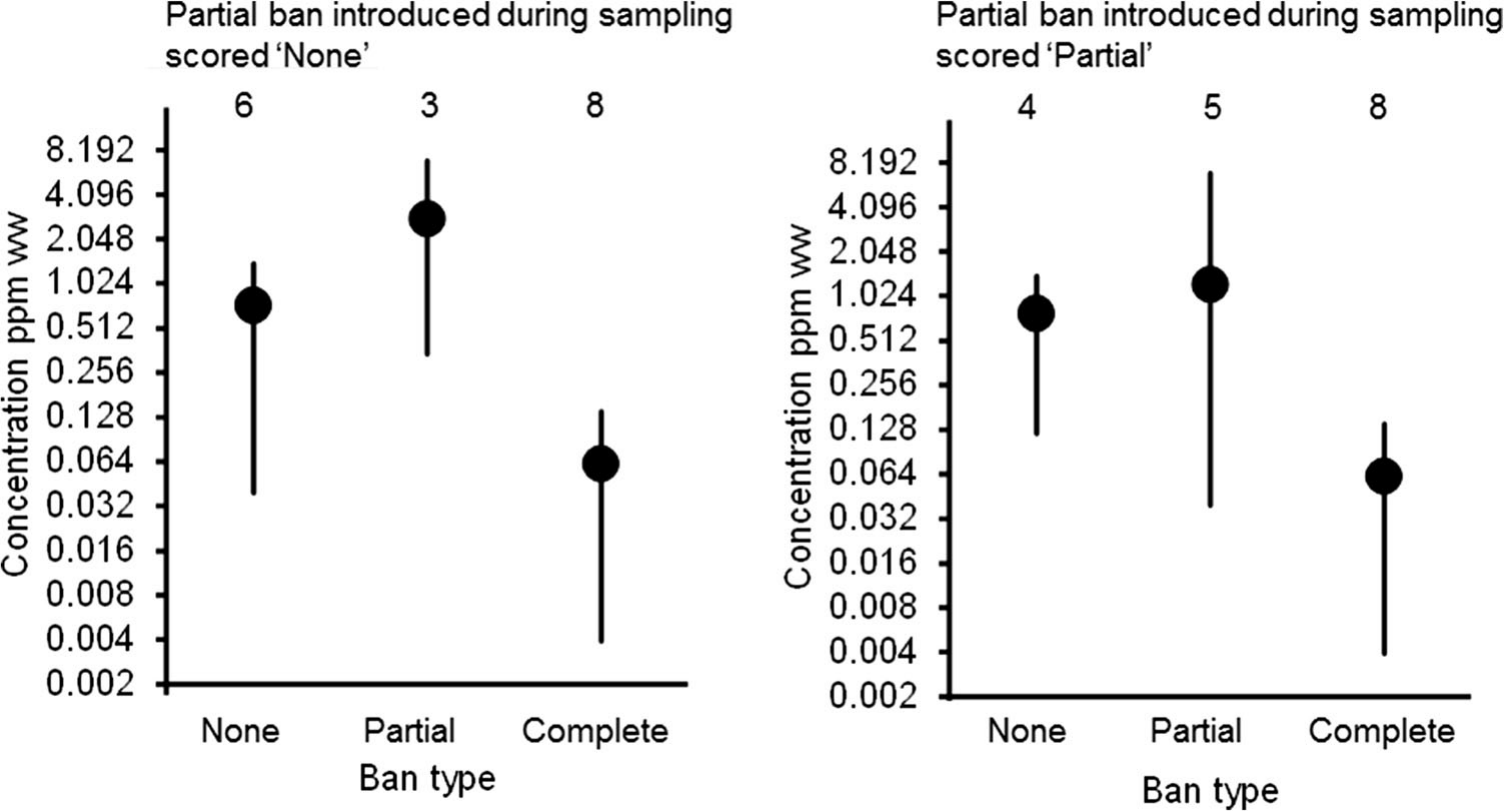
Arithmetic mean concentrations of lead (ppm w.w.) in tissues of ducks sampled where no ban on lead shotgun ammunition was in place (‘None’), where a partial ban was in place (‘Partial’) or where a complete ban was in place (Denmark only; ‘Complete’). The plotted symbol shows the grand mean for all studies combined, weighted by sample size, and the vertical line shows the range of values across studies. The number of studies contributing to the mean is shown above each point. There were two studies in which a partial ban was introduced during the sampling period. These have been treated either as having no ban (left-hand diagram) or as having a partial ban (right). The values on the logarithmic vertical axis are untransformed

Data on lead concentrations in duck muscle were available for three countries with no ban on the use of lead gunshot and for three countries with a partial ban, covering wetlands or shooting waterfowl. For two other countries, there were data on lead in duck meat for a sampling period which included the date of introduction of a partial ban. There are too few data for a formal statistical analysis, but the arithmetic mean concentration of lead in duck muscle showed no indication of being lower in countries with a partial ban than in those with no ban. This conclusion is the same regardless of whether the studies in which a partial ban came into force during the sampling period were treated as having no ban or as having a partial ban (Fig. 4). By contrast, as already described above, the arithmetic mean concentration of lead in duck muscle was consistently low in Denmark during the period when a complete ban was in place there.

**Fig. 5:**
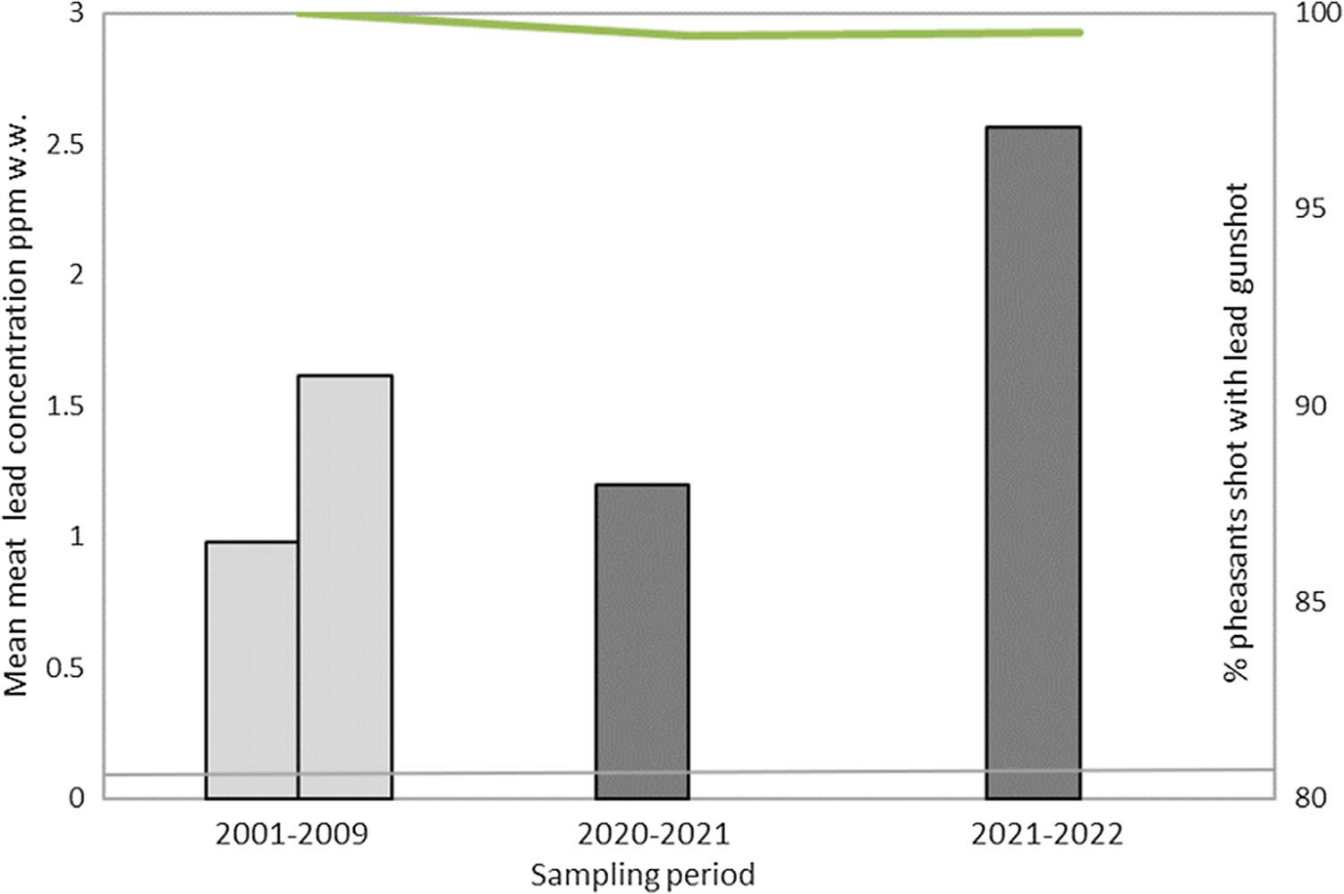
Percent of pheasants shot with lead ammunition in the UK (green line and right-hand axis) and mean lead concentrations in pheasant muscle (bars and left-hand axis) before (pale grey bars) and after (dark grey bars) the announcement by shooting organisations in February 2020 of a voluntary phasing out of lead shotgun ammunition within five years. Data on tissue lead concentrations (left vertical axis and bars) are from the following sources: (1) Pre-voluntary ban announcement; Pain et al. (2010) for the 2008–2009 hunting season (first bar, *n* = 23) and for seasons from 2001 to 2008 (second bar, *n* = 106); (2) Post-voluntary ban announcement; Wild Justice (2021) for the 2020–2021 season (*n* = 30) and 2021–2022 season (*n* = 44). Data on the proportion of pheasants shot with lead ammunition are reported in Green et al. (2021) for the 2008–2009 season (from data used in Pain et al. (2010), *n* = 10) and for the 2020–2021 season (*n* = 180), and from Green et al. 2022 for the 2021–2022 seasons (*n* = 215). The voluntary ban announcement was before the 2020–2021 season (BASC 2020). Horizontal line shows the EU Maximum Level (0.100 ppm w.w.) for lead in meats from domesticated animals

In the UK, a five-year voluntary transition to the use of non-lead gunshot for the taking of live quarry was proposed by nine major shooting organisations in February 2020 (BASC 2020). Lead concentrations in the meat of shot pheasants prior to this announcement and during the first two shooting seasons following this announcement are compared in Fig. 5, along with the results of studies that examined gunshot types used to kill pheasants in the subsequent two shooting seasons. This showed no evidence of an effect of the voluntary ban and no decrease in muscle lead concentrations in pheasants.

## Discussion

### Lead concentrations in meat from small game

Inspection of the data in Fig. 1 for countries other than Denmark, and regression analysis of data from those countries, does not indicate any marked differences in mean lead concentration among species. Although there was substantial variation in mean concentration among individual studies, this variation decreased rapidly with increasing sample size. Means did not tend to increase or decrease consistently with increasing sample size, indicating that selective publication of results based upon small samples if they gave unusually high or low mean lead concentrations probably did not occur. Variation among means for individual studies was approximately symmetrical on a logarithmic scale about the geometric mean at all sample sizes, suggesting that extreme values were probably attributable to small sample size. We, therefore, suggest that unusually high and unusually low mean values should not be excluded as outliers when calculating a grand mean value.

The high proportion of samples exceeding the EUML for meat from domesticated animals, (> 60% across Europe excluding Denmark), and the distribution of lead concentrations in gamebird meat purchased from UK retail outlets (Table 1) illustrate that high arithmetic mean concentrations result from many samples with elevated lead levels rather than a few studies with unusually high values. This, and the fact that people that frequently eat game will be exposed to a range of different concentrations throughout the year, confirms the need to include all data in the analysis. The data distribution supports the approaches taken by ECHA (2021), the FSA (2012), and other agencies that have used arithmetic mean rather than geometric mean or median concentrations in risk assessments and have included all samples rather than excluding high values as outliers.

Regression models (excluding Denmark) found that neither species nor country explained variation in mean tissue lead concentrations. Along with the decrease in variation among means with increasing sample size, this indicates that pooling all data to calculate a grand arithmetic mean, weighted by sample size, is an appropriate method to use when determining a mean value to use in human health risk assessment. At 2.422 ppm w.w., the grand arithmetic mean calculated for small game in Europe (all countries over the whole time period) is considerably greater than the arithmetic mean (upper bound) of 0.366 ppm w.w. used by ECHA (2021) for small game animals in their assessment of risks to human health from game consumption. ECHA (2021) used a mean from data provided by EFSA (2020). We cannot comment on the reasons for this disparity because the EFSA (2020) data have not been published or made available to us at the time of writing (March 2022). For this reason, we were unable to assess possible reasons for the large discrepancy between the mean concentration from our study and that of EFSA (2020). However, it is notable that ducks comprised > 50% of the EFSA (2020) sample used to calculate a mean value (Table 1–43, ECHA 2021), and these had a low mean lead concentration of 0.096 ppm w.w. in their dataset. While species differences did not explain variation among mean lead concentrations in our analysis, any anomaly associated with a mean value for a species comprising such a high proportion of the EFSA dataset would have a substantial effect on their grand mean. Ducks comprise < 10% of birds shot in the EU (Hirschfeld and Heyd 2005).

In our regression models (excluding Denmark), the only variable that appeared to explain the variation in mean tissue lead concentration was the time period within which the central year of each sampling period occurred, modelled as a categorical variable comprising three periods: 1991–2000, 2001–2010 and 2011–2021. The grand mean meat lead concentration was substantially higher in 2011–2021 than in the two earlier decades (Table 2). This resulted from a high proportion of studies having mean values that exceeded 1.0 ppm w.w. (i.e. ten times the EUML; 54% of 26 species/country combinations between 2011 and 2021 compared with 18% of 22 and 20% of 5 combinations for 2001–2010 and 1991–2000, respectively) rather than just one or two studies with extreme results; possible reasons for this are explored below. This temporal trend suggests that the most appropriate grand mean value to use for a current assessment of risk is the most recent; i.e. 5.205 ppm w.w., for 2011–2021. This is 14 times higher than that used by EFSA (2021). This large difference is likely to influence human health risk assessment.

### Possible reasons for the apparent increase in lead concentration in meat from small game animals

We found a tendency for the concentration of lead in the meat of small game animals to have increased substantially over time in those European countries with no total ban on lead gunshot use. This trend might result from a range of factors, acting individually or in combination. It is possible that some aspects of hunting technique or behaviour have changed over this period, resulting in proportionately more lead fragments occurring in the muscle tissue of small game. Awareness of the need for sustainable shooting may have resulted in more birds being shot at close range to avoid crippling, as has happened in some places for migratory waterbirds (Noer et al. 2007; Madsen et al. 2015). However, this is entirely speculative as little evidence exists as to whether such a change has happened for other species and on a wide scale, or whether shooting at closer range would necessarily result in more lead fragments in the muscle tissue of birds.

Part of the increase in muscle lead concentrations may be associated with the increased volume and availability of lead gunshot in the environment. This might have resulted in more birds ingesting lead shot and absorbing lead from the gut, and consequently higher muscle lead concentrations. While birds will primarily ingest the most available shot, usually that most recently deposited, accumulation of the approximately 20 000 tonnes of spent gunshot used every year in the EU (ECHA 2021) is likely to increase availability of spent shotgun pellets to birds in certain situations. While only a small proportion of the body burden of lead is found in muscle, it nonetheless increases with exposure, and can exceed the EUML (for meat from domesticated animals) by an order of magnitude in muscle tissue from birds that are lead-poisoned or experimentally force-fed lead gunshot (e.g. Fimreite 1984; Longcore et al.^1974^; Gasparik et al. 2012). It is, therefore, possible that increased ingestion of spent lead shotgun pellets could have contributed to the increasing trend in muscle lead concentrations. This hypothesis could be tested using long-term datasets on blood lead concentrations in live-captured birds of species that frequently ingest shot and are resident in areas with no regulatory changes. However, we are not aware of any such data. As the increase in muscle lead concentrations over time was substantial and only evident in the last decade, it appears unlikely that increased availability of lead shotgun pellets to feeding birds will have been the only explanation.

The majority of lead measured in samples of muscle is likely to result from lead fragments shot into tissue. A study of lead concentrations in the meat of UK gamebirds found that concentrations were positively correlated with both the number of shotgun pellets detected in the carcass by X-radiography and also, and independently, with the number of small fragments of lead detected (Pain et al. 2010). Since the pellets, but not the small fragments, were removed from the carcasses before analysis, this relationship is probably attributable to the contribution of small lead fragments to the concentration of lead in meat.

### The impact of risk-reduction measures on lead concentrations in game meat

Regression models indicated that variation in mean lead concentrations was best explained by the differences between Denmark and other European countries, and time period. Denmark was the only country in the dataset with a total lead gunshot ban (from 1996), and this was associated with a grand arithmetic mean tissue lead concentration for small game in Denmark that is less than one-fifth of that for other European countries (0.479 vs 2.474 ppm w.w., Table 2). However, while the ban was introduced in Denmark in 1996, there was nonetheless a decreasing trend in muscle lead concentrations over time (Figs. 2 and 3). This declining trend probably resulted largely from a campaign initiated in 2008 to raise awareness of the regulations. The campaign was stimulated by a research project in 2008 that concluded that continued illegal use of lead ammunition was the main reason for elevated lead concentrations in pheasant muscle in the early 2000s (Kanstrup 2012). Following this campaign, mean tissue lead concentrations in pheasant and woodpigeon tissue decreased substantially (Fig. 3), so it appears to have been very effective. Studies in Denmark examining the proportion of embedded shotgun pellets in pheasant and mallard carcasses (or parts of carcasses) that was composed principally of lead found this to be 16% (12/77) in pheasants and 9.5% (9/94) in mallards in 2008 (Kanstrup 2012), but this proportion fell to 1.8% (8/447) in pheasants and 2% (5/254) in mallards by 2016/2017 (Kanstrup and Balsby 2019). The trends over time in both lead concentration and shotgun pellet types are both consistent with increased compliance with the complete ban.

Another factor that may have influenced this declining trend in muscle lead concentrations in Denmark is that new generations of bismuth shot manufactured from January 2010 appeared to contain a lower percentage of lead. A bismuth shot purchased in March 2009 contained 0.315% lead and one purchased in January 2010 contained 0.095% lead (Kanstrup 2012). In Denmark, bismuth shot has been used frequently as an alternative to lead shot since the mid-1990s in particular in hunting in forests. Even shot containing < 1% lead can result in tissue muscle lead concentrations that exceed the EUML if fragments are present in analysed tissue, although the frequency with which this occurs will be much reduced with bismuth shot as compared with lead shot. Kanstrup (2012) found no evidence that steel shot was a source of lead.

In contrast to other species in Denmark, mean lead levels in duck tissue were low both before and after the awareness-raising campaign in 2008 (Fig. 3), possibly because of the long recognition by hunters of risks to waterbirds and early regulation of lead shot use for hunting in wetlands. By mid-1985, hunters’ representatives and the authorities agreed on the need for an experimental time-limited ban on lead shot for hunting at eight wetlands and from 1986 a statutory ban was in place on the use of lead shot in all Danish Ramsar sites (wetlands of international importance listed under the Convention on Wetlands—the Ramsar Convention) and on ponds used for captive-reared mallard hunting. In 1993, this was replaced by a regulation banning all use of lead shot for hunting and the trade and possession of lead shot cartridges, which became fully enforceable in 1996. Kanstrup (2019) reported that the total ban was not motivated by risks of lead poisoning to non-wetland species [or indeed reductions in risks to human health, which were not recognised at the time], but rather by the need to enforce the wetlands regulation efficiently. Habitat or site-specific regulations were considered highly inefficient, with enforcement requiring intensive policing of individual hunters’ use of non-lead shot types (Dansk Jagt 1987). The Danish total ban from 1996 appears to have been effective at substantially reducing lead gunshot use and lead poisoning of waterfowl across the whole tissue monitoring period. A side benefit of this, not recognised at the time, has been consistently low lead levels in the muscles of shot waterfowl (Fig. 3), and thus, a substantial reduction in risks to human consumers and predatory/scavenging wildlife. Kanstrup et al. (2019) found that liver lead concentrations in Danish raptors were generally below those of similar studies in other northern European countries, and none exceeded generally accepted threshold values for adverse health effects.

While the total ban on lead gunshot in Denmark was associated with a substantial reduction in lead concentrations in muscle tissue of small game animals, this does not appear to have been the case for partial (wetland or waterfowl) regulations, or voluntary bans. Excluding Denmark, many countries across Europe have partial bans on the use of lead ammunition for the shooting of waterfowl or over wetlands (Mateo and Kanstrup 2019). While some countries had partial bans in place when mean muscle tissue lead concentrations were reported, they were not lower than those from countries with no ban (Fig. 4). In addition to data presented in Fig. 4, research from Spain found that muscle lead concentrations in 24.5% of 249 duck (9 species) and 6.4% of 78 coot sampled in the Ebro delta between 2007 and 2011, after the 2001 ban on lead shot use in protected wetlands, exceeded the EUML (Mateo et al. 2014). It is highly likely that this was because of low compliance with partial bans.

Mateo et al. (2014) also found that in the Ebro delta, in ducks killed by hunters and found to contain any embedded shot, > 40% contained lead shot (lead alone or lead and steel) after the ban. Compliance increased during their study in parallel with increased enforcement of the regulations and vigilance from park rangers, and with the threat from local authorities of a hunting ban in the protected areas if non-compliance persisted. However, it is unlikely that such a high level of surveillance and enforcement would be practical in the long term or across multiple sites. Compliance has also been evaluated via a survey of hunters in Sweden, where the use of lead shot in wetlands, in practising and in competitive shooting (with a few exceptions) was banned in 2002 (Swedish Codes of Statutes, 1998). Widemo (2021) carried out a survey in 2016 of shot types used while practising and when taking game in different habitats. Results suggested that over half of the shooters practised using lead and a third used lead shot over what they considered to be wetlands, both of which contravened the legislation at that time.

The most comprehensive compliance studies have been undertaken in England where, from 1999, the use of lead gunshot has been banned by law for shooting waterfowl anywhere and also for shooting over certain wetlands (HMSO 1999). Three separate compliance studies were undertaken spanning four shooting seasons (2001/2002; 2009/2010 and 2010/2011; and 2013/2014), monitoring shot types used to kill ducks across England. Levels of compliance were consistently low, with only 32%, 30%, 30%, and 23%, respectively, of sampled ducks having been killed legally using non-lead gunshot (Cromie et al. 2015). Ongoing recent work suggests that this situation has not changed (Cromie, pers comm).

Beyond regulation, voluntary commitments have resulted from recent concern over the risks to human health and wild birds beyond wetlands. In the UK, these have included changes in policies and practices of stakeholders across a range of sectors, including commitments by game distributors and retailers to transition to non-lead ammunition (Pain et al. 2020; LAG 2022). On the 24th of February 2020, a joint statement on the future of lead gunshot for live quarry shooting in the UK (BASC 2020) was issued by nine major UK shooting and rural organisations: “*in consideration of wildlife, the environment and to ensure a market for the healthiest game products, at home and abroad, we wish to see an end to both lead and single-use plastics in ammunition used by those taking all live quarry with shotguns within five years*”. This voluntary transition has been accompanied by a range of associated activities, including awareness raising, the provision of advice, and training in the use of non-lead gunshot. However, in the first two shooting seasons following the announcement, there was no measurable reduction in the proportion of pheasants sampled from retail outlets across Great Britain that had been shot with lead ammunition: this remained at > 99% (Green et al. 2022). Figure 5 illustrates this low level of compliance and the continuously elevated mean muscle lead concentrations in pheasants purchased from food retail outlets both before and after the first two years of the voluntary ban. This accords with previous voluntary attempts to phase out the use of lead gunshot for shooting waterfowl in the UK which were also unsuccessful (Stroud 2015). This is not surprising as, alone, most voluntary schemes have limited impact and do not substitute for regulatory measures or result in substantial behavioural change (House of Lords Science and Technology Select Committee 2011; McCarthy and Morling 2015).

## Conclusions

In our review and analysis of muscle lead concentrations in small game animals, we aimed to (1) provide supporting information for an accurate evaluation of the health risks to consumers and (2) determine the effectiveness of existing voluntary and regulatory measures at reducing the amount of ammunition-derived lead in this meat.

With respect to the first objective, our study found that variation between studies in mean game meat lead concentrations decreased as sample size increased and was not explained by species nor country, except for the lower levels in Denmark, where there is a complete ban on lead gunshot. Our results suggest that for risk assessment purposes, arithmetic mean values should be used, and all data should be included, without the exclusion of unusually high or low values as outliers, to reflect year-round exposure of consumers to lead in game meat. Large sample sizes using pooled data from published studies collected using consistent methods are likely to provide a grand mean lead concentration that most closely reflects human exposure.

The review illustrated that lead derived from ammunition causes considerable contamination of game meat. Where reported, over 60% of meat samples from small game from across Europe (excluding Denmark) had lead concentrations exceeding the EUML of 0.100 ppm w.w. for meat from domesticated animals, often by more than an order of magnitude. The studies we reviewed also revealed an unexpected and unexplained increase over time in mean muscle lead concentrations in small game animals. While some of this could hypothetically result from biologically-incorporated lead (e.g. following the ingestion of lead gunshot), this pathway appears likely to contribute a relatively small amount to muscle lead concentrations in comparison to fragments from shot-in lead gunshot (e.g. Scheuhammer et al. 1998). We calculated an arithmetic mean lead concentration in small game meat of 5.205 ppm w.w. (2011–2021) from across Europe, which is fourteen times higher than that used in a recent EU-wide risk assessment.

Various restrictions on the use of lead gunshot have been introduced in European countries over the past four decades, initially to protect waterfowl health and more recently to protect the health of scavenging and predatory animals and humans (Mateo and Kanstrup 2019). With respect to our second objective, we found no evidence that partial restrictions, such as those over wetlands or for waterfowl shooting, nor calls for voluntary restraint, had resulted in a measurable reduction in lead concentrations in game meat. This was probably related to poor compliance, as previously measured in studies in which the composition of shot used to kill birds had been identified. In contrast, the total ban on lead gunshot use introduced in 1996 in Denmark was associated with mean lead concentrations in duck meat consistently below the EUML for other meats, and similarly low concentrations in other gamebirds subsequent to an information campaign highlighting the regulations in 2008 (Kanstrup 2012).

Combined, these results suggest that a total ban on the placing on the market and use of lead ammunition is likely to be necessary to ensure that levels of lead in the meat of wild small game animals fall below the EUML for other meats and minimise risks to human health. Surveillance and hunter awareness-raising activities will be necessary to ensure compliance (Widemo 2021), particularly directly following any restriction. The setting of a formal EUML for game meat in line with that used for meat from domestic animals (0.100 ppm w.w.) would strengthen national reporting requirements, facilitate compliance monitoring, standardise food safety requirements across the EU and help reduce risks to human health (Thomas et al. 2020).

## Supplementary Information

Below is the link to the electronic supplementary material.

Supplementary file1 (PDF 764 kb)
