# Supplementary material for: How contaminated with ammunition-derived lead is meat from European small game animals? Assessing and reducing risks to human health
Source: Ambio. 2022 May 10;51(8):1772–85. doi: 10.1007/s13280-022-01737-9 (PMC9200912; doi:10.1007/s13280-022-01737-9)
Supplement: Supplementary file 1 — Supplementary file1 (PDF 764 kb) [file 13280_2022_1737_MOESM1_ESM.pdf]

Ambio

Supplementary Information
This supplementary information has not been peer reviewed.

Title: **How contaminated with ammunition-derived lead is meat from European small game animals? Assessing and reducing risks to human health.**

SI Appendix 1

Literature Review methods and data identified

We followed ROSES (RepOrting standards for Systematic Evidence Syntheses) search methods where appropriate to review the literature and identify relevant publications.

Background: The rational for our review was to identify papers that reported arithmetic mean lead concentrations in the muscle tissue of wild small game animals (gamebirds, rabbits and hares) killed using shotgun ammunition in Europe.

Objective: To enable a comparison to be made of the concentration of lead in meat across species of game animal, countries and time periods, and in countries with and without legal restrictions on the use of lead gunshot. We also aimed to use the results to identify the most appropriate way of calculating a mean lead concentration for small game for human health risk assessment purposes and to identify the effect of risk reduction measures on lead concentrations in game meat.

Search Strategy:

(1) We searched the literature using the web-based search engine Google Scholar https://scholar.google.co.uk

Dates: We confined our search to papers published between 1990 and 2022.

Topic Words: We searched using the following topic words: combinations of the terms 'muscle' and 'lead' and either: 'game' or 'bird' or 'mammal' or 'gamebird' or 'pheasant' or 'partridge' or 'duck' or 'goose' or 'geese' or 'wader' or 'plover' or 'snipe' or 'redshank' or 'thrush' or 'migratory bird' or 'rabbit' or 'hare'. We repeated the search replacing 'muscle' by 'tissue' and then by 'meat', and 'lead' by 'heavy metal'. We performed additional searches using the topic words: national surveillance, metals, risk assessment, food, Europe, shot, fragments.

When large numbers of articles were found, we examined abstracts of the first one hundred potentially relevant articles (by which time no additional relevant information was found), or until there were no longer any obvious relevant articles.

(2) We further examined references cited by the authors of papers identified in (1) above.
(3) We examined papers cited by six national assessments of risks to human health from the consumption of wild game, as cited in the main text of our paper, and also in the European Chemical Agency (ECHA) 2021 restriction proposal (ECHA 2021a).
(4) We used two datasets from the grey literature in the UK that one or more of the studies' authors had been involved in. We present the data collection methods and chemical analysis in full in SI Appendix 2 for quality control to ensure comparability with peer-reviewed publications.
(5) Data from the UK and Denmark was particularly relevant as we wished to compare the effects of a total statutory ban on the use of lead gunshot, as is present in Denmark, and a voluntary ban on lead gunshot use for hunting, as is present in the UK, on mean lead concentrations in game meat. We searched national surveillance data for Denmark on the Danish Food and Veterinary Administration website:
https://www.foedevarestyrelsen.dk/Kontrol/Kontrolresultater/Sider/Tungmetaller.aspx
There is also a statutory ban on the use of lead gunshot in the Netherlands, but we were unable to find studies of lead concentration in small game animal tissue for that country. We

searched for UK surveillance data on the Veterinary Medicines Directorate current and archived websites:
Current reports:
https://www.gov.uk/government/collections/residues-statutory-and-non-statutory-surveillance-results
Archived reports:
https://webarchive.nationalarchives.gov.uk/ukgwa/20140909095919/http://www.vmd.defra.gov.uk/VRC/reports/surveillance.html

Inclusion criteria:

We included data that met all of the following criteria:

- The sample size and arithmetic mean concentration of lead in muscle tissue of individual species/species groups was provided or could be calculated (e.g., from raw data). Ideally, we would have analysed raw data consisting of concentrations for individual animals, but such data were hardly ever available. Neither was it possible to consistently obtain the variance of concentrations across individual animals in the sample. We therefore extracted the arithmetic mean value and recorded the sample size (number of animals sampled) as a proxy for the likely precision of the estimated mean.
- The game species had been shot in the wild.
- We could ascertain whether concentrations were given as wet or dry weight values.
- The sampling country was given.
- The date of sampling was given or could reasonably be inferred (see below).
- Information was available on analytical procedures including limits of detection/quantification (LOD/LOQ).

When this information was available data were extracted and treated as follows:

Information was extracted on species, country, sampling date, source of birds and harvesting method, shot type used (where reported), whether shot were removed prior to lead analysis, arithmetic mean lead concentrations, proportion of samples exceeding a lead concentration of 0.100 ppm w.w. (the EUML).

To ensure consistency in our dataset, we treated the data as follows:

- If lead concentrations were presented for more than one muscle in the animal (i.e., breast and leg separately) a mean value was calculated. Where data were presented separately for each breast of a bird, the mean of the two breast samples was used, so that total sample sizes represented numbers of birds rather than numbers of sub-samples.
- The vast majority of studies presented data in parts per million (ppm) wet weight (w.w.), which is equivalent to mg kg^{-1}. Where results were presented in ppm dry weight (d.w.) we accepted the conversion factor to wet weight cited by the authors or otherwise used a conversion factor of 1 ppm w.w. ≈ 3.2 ppm d.w., as used by Mateo et al. (2014).
- Where raw data were available, in order to calculate arithmetic means we assumed that samples with concentrations listed as below the limit of detection/limit of quantification (LOD/LOQ) had concentrations of half these limits (unless stated otherwise). We treated Roselli et al. (2016) differently as the minimum detectable concentration (MDC) was high at 0.326 mg/kg w.w. For this study we excluded data on woodpigeons as all samples contained <0.326 ppm, and 50% of this would nonetheless have been greater than the EUML. We include data from woodcock (n = 4) from this study and used 50% of the MDC for one sample as lead

concentrations in the other three samples considerably exceeded the MDC (at 0.94, 5.78 and 8.90 ppm w.w.), thus the fourth value had little impact on the arithmetic mean.

- In one study (Stamberov et al., 2018) authors did not mention whether wet or dry weights had been presented. We assumed these to be wet weights as sample drying was not described in the methods and permissible levels described in the text appeared in line with wet weights.
- Samples from the majority of studies were analysed as raw meat, but in several studies samples were cooked using standard methods and recipes prior to analysis to simulate human exposure (Pain et al. 2010; Mateo et al 2011; Carpene et al. 2020). In Carpene et al. (2020), while meat from pheasants and hares used in recipes was muscle tissue from eviscerated animals, the muscle of woodcock (n = 5) was mixed with other soft meat from the birds, which is standard cooking practice for woodcock.
- For five of 23 EU studies (excluding statutory surveillance) the date of sampling of small game was not given. For the remaining studies, the median time that elapsed between sampling and publication was 3 years. We therefore assumed that sampling took place three years before the publication date in the 5 studies for which sampling dates were not given.

Reasons for data exclusion:

Data from papers that did not meet our inclusion criteria were rejected. For example, in a few papers,concentrations were not presented as arithmetic means and/or arithmetic means could not be calculated (e.g., Mateo et al. 2014 – cited in the main reference list).

Data from the UK surveillance scheme (VMD various) were excluded from our analyses because of an evident anomaly. We examined an annual 20-year time series of the proportion of pheasants and partridges with muscle lead concentrations greater than the EUML for the period 2001-2020. There was an unexplained stepwise decrease between 2007 and 2008, with proportions up to 2007 being high and similar both to those from other UK studies and European studies for pheasants and partridges. From 2008 onwards the proportions were uniformly near zero. VMD substantially changed their reporting threshold in 2008, and results from subsequent surveillance were dramatically different to those from previous surveillance, despite there being no changes in UK shooting methods or cartridge types used between the sampling periods (see Green et al., 2021; 2022). Other sources of data from the UK provided broadly consistent results over time (Pain et al., 2010; Avery, 2016; Wild Justice, 2022). We have consulted VMD about this apparent anomaly, which we suspect may be related to the source(s) of pheasants and partridges. However, we have not been able to resolve the issues and so we have excluded the VMD surveillance data from our analyses.

The following studies met our search criteria and contained data used in our analysis

Avery 2016. Lead Week, 12#leadweekmia https://markavery.info/2016/01/27/lead-week-12-pbweekmia/

Carpenè, E., Andreani, G., Ferlizza,E., Menotta, S., Fedrizzi, G., and Isani, G. 2020. Trace Elements in Home-Processed Food Obtained from Unconventional Animals. *Life* 10 (5): 75. https://doi.org/10.3390/life10050075

Demirbas, Y.; Erduran, N. 2017. Concentration of Selected Heavy Metals in Brown Hare (*Lepus Europaeus*) and Wild Boar (*Sus Scrofa*) From Central Turkey. *Balkan Journal of Wildlife Res*earch 2017, 4, 26–33. DOI: 10.15679/bjwr.v4i2.54

Długaszek, M. and Kopczyński, K. 2013. Elemental Composition of Muscle Tissue of Wild Animals from Central Region of Poland. *International Journal of Environmental Res*earch 7(4):973-978. https://dx.doi.org/10.22059/ijer.2013.680

Doganoc, D. Z. & GačKnic, K. Ŝ. 1995. Lead and Cadmium in Meat and Organs of Game in Slovenia. *Bulletin of Environmental Contamination & Toxicology* 54:166-170. https://doi.org/10.1007/BF00196284

Drápal, J., Ruprich, J. & Šťastný, K. Undated. Lead contamination of wild game meat from the viewpoint of veterinary supervision. Maso-interational.cz http://www.maso-international.cz/download/39_42.pdf .

DVFA various. Danish Food and Veterinary Administration. Results from food studies for heavy metal content. Ministeriet for Fødevarer, Landbrug og Fiskeri Fødevarestyrelsen https://www.foedevarestyrelsen.dk/Kontrol/Kontrolresultater/Sider/Tungmetaller.aspx .

Dżugan, M., Zielińska, S., Hęclik, J., Pieniążek, M. & Szostek, M. 2012. Evaluation of heavy metals environmental contamination based on their concentrations in tissues of wild pheasant (*Phasianus colchicus l*.) *Journal of Microbiology, Biotechnology and Food Sciences* 2 (1): 238-245. https://www.jmbfs.org/jmbfs-dzugan-b/?issue_id=1307&article_id=16

Ertl, K., Kitzer, R. & Goessler, W. 2016. Elemental composition of game meat from Austria, *Food Additives & Contaminants:* Part B, 9:2, 120-126, DOI:10.1080/19393210.2016.1151464 https://www.tandfonline.com/doi/full/10.1080/19393210.2016.1151464 .

Flis, M., Grela, E.R., Żukowska, G. & Gugała, D. 2020. Nutritional composition and heavy metal content in breast and thigh muscles of wild and intensively reared common pheasants (*Phasianus colchicus*). *Journal of Veterinary Research* 64: 305-312. https://doi.org/10.2478/jvetres-2020-0028

Gasparik, J., Vladarova, D., Capcarova, M., Smehyl, P., Slamecka, J., Garaj, P., Stawarz, R., & Massanyi, P. 2010. Concentration of lead, cadmium, mercury and arsenic in leg skeletal muscles of three species of wild birds. *Journal of Environmental Science and Health*, Part A, 45(7), 818–823. https://doi.org/10.1080/10934521003708992 .

Halecki, W., l Gasiorek, M., Wajdzik, M., Pajak, M. & Kulak, D. 2017. Population parameters including breeding season of the European brown hare (*Lepus Europaeus*) exposed to cadmium and lead pollution. *Fresenius Environmental* 26 (4): 2998-3004.

Kalisińska, E., Salicki, W., Mysłek, P., Kavetska, K.M. & Jackowski, A. 2004. Using the Mallard to biomonitor heavy metal contamination of wetlands in north-western Poland. *Science of the Total Environment* 320: 145–161. https://doi.org/10.1016/j.scitotenv.2003.08.014

Koréneková, B., Mkalická, M., Kožárová, I., Nagy, J., Máté, D. & Naď, P. 2008. Comparison of cadmium, lead and nickel accumulation in liver, breast and leg muscles of pheasants. *Slovak Journal of Animal Science* 41 (4): 184 – 186. https://sjas.ojs.sk/sjas/article/view/452

Lucia, M., André, JM., Gontier, K. et al. 2010.Trace Element Concentrations (Mercury, Cadmium, Copper, Zinc, Lead, Aluminium, Nickel, Arsenic, and Selenium) in Some Aquatic Birds of the Southwest Atlantic Coast of France. *Archives of Environmental Contamination and Toxicology* 58: 844–853. https://doi.org/10.1007/s00244-009-9393-9.

Mateo, R., Baos, A.R., Vidal, D., Camarero,P.R., Martinez-Haro,M. & Taggart, M. A. 2011. Bioaccessibility of Pb from Ammunition in Game Meat Is Affected by Cooking Treatment. *PLoS One* https://doi.org/10.1371/journal.pone.0015892.

Myslek, O. & Kalisińska, E. 2006. Contents of selected heavy metals in the liver, kidneys and abdominal muscle of Brown Hare (Lepus Europeaus, Pallas, 1778) in central Pomerania, Poland. *Polish Journal of Veterinary Sciences* 9(1):31-40.

Pain, D.J., Cromie, R.L., Newth, J., Brown, M.J., Crutcher, E., Hardman, L.H., Hurst, L., Mateo, R., Meharg, A.A., Moran, A.C., Raab, A., Taggart, M.A. & Green, R.E. 2010. Potential Hazard to Human Health from Exposure to Fragments of Lead Bullets and Shot in the Tissues of Game Animals. *PLoS ONE* 5(4):e10315. https://doi.org/10.1371/journal.pone.0010315 .

Pilarczyk, B., Tomza-Marciniak, A., Pilarczyk, R., Udała, J., Kruzhel, B., & Ligocki, M. 2020. Content of essential and non-essential elements in wild animals from western Ukraine and the health risks associated with meat and liver consumption. *Chemosphere* 244: 125506. https://doi.org/10.1016/j.chemosphere.2019.125506.

Roselli, C.; Desideri, D.; Meli, M.A.; Fagiolino, I.; Feduzi, L. 2016. Essential and toxic elements in meat of wild birds. *Journal of Toxicology and Environmental Heal*th Part A 79: 1008–1014. https://doi.org/10.1080/15287394.2016.1216490

Sevillano-Morales, J.S., Sevillano-Caño, J., Cámara-Martos, F. et al. 2021. Risk Assessment of Cd, Cu, and Pb from the consumption of hunted meat: red-legged partridge and wild rabbit. *Biological Trace Element Research* 199: 1843–1854. https://doi.org/10.1007/s12011-020-02290-w .

Sevillano-Caño, J.,Cámara-Martos, F., Zamora-Díaz, R., Sevillano–Morales, J.S. 2021. Lead concentration in game migratory upland bird meat: Influence of ammunition impacts and health risk assessment. *Food Control* 124: 107835. https://doi.org/10.1016/j.foodcont.2020.107835.

Stamberov, P., Zhelev, C., Todorov, T., Ivanova, S., Mehmedov, S., Manev, I., & Taneva, E. 2018. Epidemiological data on lead tissue concentration in game birds induced by lead pellets. *Sciendo* 1(1), 479–484. DOI: 10.2478/alife-2018-0075.

Szymczyk, K. & Zalewski, K. 2003. Copper, Zinc, Lead and Cadmium Content in Liver and Muscles of Mallards (Anas Platyrhychnos) and Other Hunting Fowl Species in Warmia and Mazury in 1999-2000. *Polish Journal of Environmental Studies* 12(3): 381-386. http://www.pjoes.com/Copper-Zinc-Lead-and-Cadmium-Content-in-Liver-and-Muscles-of-Mallards-Anas-Platyrhychnos,87570,0,2.html

Wild Justice 2021. High lead levels in Waitrose and Harrods game meat. 18th December 2021. https://wildjustice.org.uk/lead-ammunition/high-lead-levels-in-waitrose-and-harrods-game-meat/

SI Appendix 2

Sample collection, preparation and analysis of UK red grouse and pheasant meat

Red grouse: 40 fresh red grouse carcasses were purchased (in packs of two, with each individual grouse in a sealed plastic bag) vacuum packed from a food retail supermarket chain in September 2015. Grouse were purchased from three separate stores of the same chain in England. The carcasses were prepared by boiling in the bag in accordance with cooking instructions on the packet. After cooking, all muscle meat that could readily be removed from the carcass was taken and inspected visually for the presence of shot and any shot found were removed and recorded. Meat was subsequently sent frozen in sealed food grade bags to the Environmental Research Institute (ERI), University of the Highlands and Islands. X-radiography to identify any additional shot was performed, which were recorded and removed prior to chemical analysis.

Pheasants: 75 fresh whole pheasant carcasses or pheasant breasts were purchased from two UK food retail supermarket chains and one department store that sells food (Outlets 1-3). These were different to the supermarket chain from which red grouse were purchased in 2015. In 2020, 10 pheasant breasts were purchased from Outlet 1 and 20 whole carcasses from Outlet 2. In 2021, 16 pheasant breasts were purchased from Outlet 1; 14 from Outlet 2 and 15 whole carcasses from Outlet 3. Sub-samples of muscle tissue were taken from each pheasant/breast. For whole carcasses, a sample was taken from each breast and each leg, from the same general area each time without either avoiding or targeting areas that looked damaged by shot. For breasts, three or four sub-samples were taken from whole breast fillets from the same general area each time, towards the corners, but avoiding the edge of the meat. Again, damaged tissue was neither chosen nor avoided. Samples were packaged in sealed food grade plastic bags and sent frozen to ERI for analysis. Shot observed were recorded and removed prior to analysis.

Chemical analysis:

Red grouse: Cooked meat was weighed, with samples from individual grouse ranging from 93-196g (mean of 151.3g) wet weight. The meat samples were then dried in an oven (at 65°C) to constant dry weight and then placed in a desiccator to cool. The moisture content of the meat was calculated to allow Pb data to be calculated per unit dry or wet weight. Each sample was then milled individually in a food grade coffee bean/spice mill to a fine powder. The mill was cleaned thoroughly between samples, by using dry laboratory tissue and a stream of compressed high-pressure air to remove all dry residual particles of material. Each milled sample was stored in a food grade zip-lock bag, then sub-sampled, taking 1g for subsequent total acid digestion. These sub-samples were digested in an open heating block system, using acid cleaned pure quartz test tubes and trace metal grade concentrated nitric acid and hydrogen peroxide. Digested samples were made up to 14ml total volume in PP (polypropylene) tubes with Milli-Q water and analysed for Pb at 220.353nm using ICP-OES (inductively coupled plasma optical emission spectroscopy; using a Varian 720-ES instrument). Procedural blanks (1ml of Milli-Q water treated as if a sample) and a certified reference material (CRM NIST 8414 - bovine muscle) were also analysed alongside the grouse samples for quality control/assurance purposes. All grouse samples provided lead data above the limit of detection for the process (0.047 mg/kg Pb in tissue, by wet weight) and data obtained for the bovine muscle CRM was within tolerance (CRM certified lead level = 0.38 +/- 0.24 mg/kg; data obtained during study = 0.31 +/- 0.08 (n = 4)).

Pheasants: Samples were tested as above for grouse, but with some slight modifications. The frozen samples were again dried in a drying oven at 65°C to constant dry weight; with the average wet weight sample size being 38.3g, dropping to 11.8g after drying – resulting in an average moisture content of

69%. Dried samples were then milled to a fine powder (as above) and stored in zip-lock bags in a desiccator post mill. Sub-samples (0.4g) were then digested using a microwave digestion system (Anton Paar, Multiwave PRO) and pressurised Teflon vessels – again using trace metal grade nitric acid and hydrogen peroxide. Following complete dissolution solutions were made up to 16ml final volume with Milli-Q water. In parallel with samples, procedural blanks were analysed in each microwave digest batch (of 24 digests) to attain a procedural limit of detection for the process, and a certified reference material (CRM) was used to ensure Pb recovery was within tolerance (strawberry leaf powder (LGC 7162); with a certified Pb level of 1.8 +/-0.4 mg/kg dry weight). Once digestions were complete, all samples/CRMs/blanks were analysed using an ICP-OES system (an Agilent 5900 instrument). The 220.353nm Pb line was again used for final data processing/calculations and CRM recovery was within the above CRM tolerance.

One pheasant sample was found during analysis to contain a shotgun pellet that had been missed during sample preparation. The concentration of lead was 3,114 ppm w.w. and this sample was excluded from analysis of mean lead concentrations as whole/near whole shotgun pellets would probably be removed before or during eating. The sample size for pheasants was thus 74.

SI Appendix 3.

A comparison of mean lead concentrations in the meat of small game according to measures restricting the use of lead ammunition in Europe

A number of countries across Europe have introduced regulatory measures restricting the use of lead gunshot over the last 35 years, largely for shooting waterfowl and/or over wetlands. These were reviewed by Mateo & Kanstrup (2019) and are reported as part of national reporting under the African Eurasian Waterbird Agreement (e.g., see AEWA 2018). We compare mean lead concentrations in muscle tissue from studies of ducks shot in different European countries: (1) with no lead gunshot restrictions in place, (2) with partial restrictions in place, covering the shooting of wildfowl and/or over wetlands, (3) with a total ban on the use of lead gunshot for all shooting (i.e., Denmark) at the time of sampling.

In Denmark, since the early 1980s a number of voluntary and statutory bans on the use of lead gunshot have been introduced to protect wildfowl from lead poisoning, culminating in a complete ban on the use of lead gunshot for all shooting in 1996 combined with restriction on its trade and possession (Kanstrup 2019). Elevated (>EUML) tissue lead concentrations continued to be reported after 1996 for some species, including pheasant, and in 2008 a research project was initiated to identify the source (Kanstrup 2012). This project found low lead concentrations (arithmetic mean of 0.030 ppm w.w.) in 30 pheasants shot under controlled conditions with non-lead ammunition in areas in which elevated lead concentrations had previously been found. The project concluded that these elevated levels resulted largely from continued illegal use of lead gunshot and a campaign was instigated to raise awareness of the regulations on lead shot (Kanstrup 2012). We examine data collected as part of statutory surveillance (available from 2004) on tissue (muscle) lead levels in pheasants, duck and woodpigeons before and after the 2008 awareness campaign to evaluate its impact.

In the UK, a five-year voluntary transition to the use of non-lead gunshot for the taking of live quarry was announced by major shooting organisations in February 2020. We compare lead concentrations in pheasant meat purchased from retail outlets both before and after this announcement and compare this with data evaluating compliance with the voluntary ban (Cromie et al. 2015; Green et al. 2022).

References

AEWA. 2018. 7th Session of the Meeting of the Parties (MOP7). National Reports. https://www.unep-aewa.org/en/documents/national-reports

AEWA. 2018. 7th Session of the Meeting of the Parties (MOP7). National Reports. https://www.unep-aewa.org/en/documents/national-reports

Cromie, R., Newth, J., Reeves, J., O'Brien, M. et al. 2015. "The sociological and political aspects of reducing lead poisoning from ammunition in the UK: why the transition to non-toxic ammunition is so difficult". In: Delahay R.J., Spray, C.J. (Eds) Proceedings of the Oxford Lead Symposium: Lead ammunition: understanding and minimizing the risks to human and environmental health. Edward Grey Institute: Oxford University, pp 104–124. http://www.oxfordleadsymposium.info/wpcontent/uploads/OLSproceedings/papers/OLSproceedingscromie_newth_reeves_obrien_beckman_brown.pdf

Green, R.E., Taggart, M.A., Pain, D.J., Clark, N.A., Clewley, L., Cromie, R., Dodd, S.G., Elliot, B., Green, R.M.W., Huntley, B., Huntley, J., Pap., S., Leslie, R., Porter, R., Robinson, J., Sheldon, R., Smith, KW., Smith, L., Spencer, J. & Stroud, D. 2022. Effectiveness of actions intended to achieve a voluntary

transition from the use of lead to non-lead shotgun ammunition for hunting. Conservation Evidence 19: 8-14. https://doi.org/10.52201/CEJ19/SAFD88

Kanstrup, N. 2012. Lead in game birds in Denmark: Levels and sources. - Danish Academy of Hunting. Article, 2012-02-1. jaegerforbundet.dk.

Kanstrup, N. 2019. Lessons learned from 33 years of lead shot regulation in Denmark. Ambio 48: 999-1008. https://doi.org/10.1007/s13280-018-1125-9

Mateo, R. & Kanstrup, N., 2019. Regulations on lead ammunition adopted in Europe and evidence of compliance. Ambio 2019, 48:989–998
https://doi.org/10.1007/s13280-019-01170-5

SI Appendix 4

Statistical Methods

We used weighted ordinary least squares regression models to examine the relationships between mean tissue lead concentrations and explanatory variables. The dependent variable in all models was the loge-transformed arithmetic mean lead concentration for a given species from a single study. We conducted the modelling in two phases. In the first phase, we excluded results from Denmark because it was the only European country for which data were available where a complete ban on the use of lead gunshot for hunting was in place during the period when data on lead concentrations were available. This ban was fully enforceable from 1996, covering most of our sampling period and the whole period for which tissue lead concentrations were available from Denmark. Each log-transformed mean concentration was weighted by w = ni*N/NS, where ni is the number of samples contributing to that mean, N is the number of means (53) and NS is the total number of samples across all species-country combinations (1342). Hence, the weights sum to N. The explanatory variables considered were country (categorical variable with 16 levels), species or species group (categorical variable with 13 levels) and decade (categorical variable with 3 levels for 1991-2000, 2001-2010 and 2011-2021). We fitted eight models which included all possible combinations of the main effects of these three variables and also the null model with no explanatory variables. Values of ΔAICc and AICc weights (Burnham & Anderson 2002) were compared across the eight models.

Based upon the results of the first phase of the analysis (given later in the Results section), we fitted weighted ordinary least squares regression models to examine the relationship between mean tissue lead concentrations and the following explanatory variables: species or species group (categorical variable with 13 levels); whether the study was from Denmark or not (binary variable; yes=1, no=0); the central calendar year of the sampling time period (continuous variable) and the two-way interaction term between Denmark or not and central year. Weighting of species-country means was as described for the first phase, except that the number of means N was 76 and the total number of samples NS across all species-country combinations was (1827). The model set included eight models which included all possible combinations of the main effects of these three variables and the null model with no explanatory variables. In addition, we fitted models with Denmark or not, central year, and the interaction term of these two variables, with and without the effect of species. Hence, a total of ten models were fitted. Values of ΔAICc and AICc weights were compared across the ten models.

We calculated grand arithmetic means, weighted for sample sizes, with 95% bootstrap confidence intervals. The bootstrap procedure involved taking 10,000 bootstrap samples for the observed data by selecting results from individual studies at random, with replacement, until the total number of individual animals included in the bootstrap sample just exceeded the actual grant total. This procedure resulted in approximately similar NS values for all bootstrap samples. This was done for all data combined, for data excluding Denmark and for Denmark alone separately, and for three time periods: 1990-2000; 2001-2010; 2011-2021. We also wished to calculate the grand arithmetic mean lead concentration for the whole of Europe. To do this, we needed to combine the mean for Denmark, which had total lead gunshot ban thorough the period for which we had data, with the mean for countries without a complete ban. However, a disproportionately large number of samples have been analysed from Denmark, compared with the amount of game shooting in that country. Data on the number of hunters in each European country in 2010 from FACE (2010) show that 2.6% of European hunters were in countries with a complete ban on lead gunshot (Denmark and the Netherlands). We assumed that the mean concentration for Denmark applied to both of these countries and assumed that 2.6% of shooting of small game was in complete ban countries and 97.4% was in countries without

a complete ban. We used these proportions to calculate a weighted arithmetic mean concentration for countries with and without complete bans combined.

References

Burnham, K.P. & Anderson, D.R. 2002. Model Selection and Multimodel Inference. Second Edition.Springer: New York, USA.

FACE 2010. Numbers of Hunters. Annual Report 2009-2010. P 16.

Appendix 5.

Data Used (Codes given below)

COUNTRY CODE	SPECIES CODE	PERIOD CODE	N	MEAN PB CONC. PPM W.W.	STUDY CODE
7	1	3	8	0.137	1
7	4	3	5	0.943	1
7	8	3	6	3.395	1
9	1	3	20	0.297	2
11	1	2	7	0.482	3
11	1	2	68	0.3286	4
11	10	2	68	0.3545	4
11	11	2	24	0.2389	4
3	1	2	65	0.708	5
3	8	2	21	0.017	5
3	10	2	60	0.546	5
1	1	3	10	125	6
1	8	3	9	9	6
7	1	3	1	1.78	7
17	4	3	4	3.95	7
10	5	3	1	98.5	7
8	6	3	1	33.4	7
8	7	3	2	0.94	7
6	10	3	1	0.54	7
2	1	3	10	0.351	8
2	2	3	10	0.337	8
2	5	3	10	0.968	8
2	6	3	10	4.6	8
2	10	3	10	6.942	8
9	1	1	7	0.075	9
9	10	1	47	0.12	9
9	1	2	6	0.007	10
13	2	2	64	2.55	11
13	2	3	89	1.278	12
13	9	3	74	0.978	12
13	3	3	27	3.41	13
13	6	3	21	0.98	13
13	7	3	35	0.82	13
14	8	3	15	7.83	14
16	8	3	8	0.691	15
9	8	1	64	0.28	16
9	8	2	60	0.183	17
9	8	2	11	0.07	18
15	1	3	74	2.01	19
15	1	2	23	0.98	20
15	2	2	26	1.12	20

15	4	2	16	3.411	20
15	3	2	21	0.433	20
15	12	2	20	1.165	20
15	10	2	16	0.341	20
15	12	3	40	10.79	21
4	1	2	264	0.8102	22
4	1	2	29	0.023	22
4	1	3	16	0.012	22
4	1	3	7	0.0015	22
4	1	3	4	0.16	22
4	1	3	4	0.017	22
4	1	3	4	0.0081	22
4	1	3	3	0.008	22
4	10	2	12	0.069	22
4	10	3	16	0.084	22
4	10	3	13	0.03	22
4	10	3	4	0.033	22
4	10	3	4	0.14	22
4	10	3	2	0.0039	22
4	10	3	2	0.055	22
4	10	2	39	0.059	22
4	3	2	32	0.332	22
4	3	2	4	0.0072	22
4	3	3	10	0.002	22
4	3	3	5	0.0072	22
4	3	3	3	0.0651	22
4	3	3	4	0.0018	22
4	3	3	4	0.0086	22
12	10	1	35	0.778	23
9	10	1	97	1.396	24
5	10	2	2	0.0391	25
5	13	2	3	0.0438	25
5	13	2	8	0.2438	25
5	13	2	1	0.19	25
5	13	2	1	0.04	25

**Species**	**Code**
Pheasant	1
Partridge (Grey & red-legged)	2
Woodpigeon	3
Woodcock	4
Common Quail	5
Turtle Dove	6

Thrush spp.	7
European Hare	8
European Rabbit	9
Duck spp	10
Coot	11
Red Grouse	12
Coastal shorebird	13

**Country**	**Country code**
Austria	1
Bulgaria	2
Czech Republic	3
Denmark	4
France	5
Hungary	6
Italy	7
Italy & Greece	8
Poland	9
Romania	10
Slovakia	11
Slovenia	12
Spain	13
Turkey	14
UK	15
Western Ukraine	16
Various EU countries	17

**Period code**	**Middle date in this period**
1	1991-2000
2	2001-2010
3	2011-2021

**Study Code**	**Reference (SI Appendix 1 Reference list)**
1	Carpene et al. 2020.
2	Flis et al. 2020.
3	Koréneková et al. 2008
4	Gasparik et al. 2010
5	Drapal et al. (undated)
6	Ertl et al. 2016
7	Roselli et al. 2016
8	Stamberov et al. 2018
9	Szymczyk & Zalewski 2003
10	Dzugan et al.2012.

11	Mateo et al. 2011
12	Sevillano-Morales et al. 2021
13	Sevillano-Caño et al. 2021
14	Demirbaş & Erduran 2017
15	Pilarczyk et al. 2020
16	Myslek & Kalsinska 2006
17	Halecki et al. 2017
18	Długaszek & Kopczyński 2013
19	Wild Justice
20	Pain et al. 2010
21	Avery 2016
22	DVFA various
23	Doganoc & Gacnik 1995
24	Kalisinska et al. 2004
25	Lucia et al. 2010
